# Tissue Cadherin 17 (CDH17): An Important Prognostic Determinant of Colorectal Cancer Using Digital Image Analysis

**DOI:** 10.1002/cnr2.70069

**Published:** 2024-12-01

**Authors:** Lui Ng, Wing Shan Yu, Nilar Myat Aung, Pauline Leung, John Moon Luk, Dennis Anthony Wong, Stella Sun, Dominic Chi‐Chung Foo

**Affiliations:** ^1^ Department of Surgery School of Clinical Medicine, Li Ka Shing Faculty of Medicine, The University of Hong Kong Pokfulam Hong Kong SAR China; ^2^ Tiberias Technology (HK) Limited Shatin Hong Kong SAR China; ^3^ Arbele Limited Shatin Hong Kong SAR China; ^4^ Arbele Corp Seattle Washington USA

**Keywords:** automated immunohistochemistry, cadherin 17, colorectal cancer, prognosis

## Abstract

**Background:**

Colorectal cancer (CRC) is a commonly diagnosed malignancy with significant mortality rates worldwide. The identification of robust prognostic biomarkers for prediction of survival outcomes and recurrence can aid disease management and improve patients' quality of living.

**Aim:**

This study aimed to investigate the prognostic value of cadherin 17 (CDH17) tissue expression in CRC patients by utilizing a standardized automated immunohistochemistry (IHC) platform integrated with a digitalized scoring system.

**Methods and Results:**

IHC was conducted to assess CDH17 expression on tumor tissues obtained from 150 retrospective CRC cases. A computer‐assisted imaging analysis was performed to quantify CDH17 tissue expression using an IHC scoring algorithm known as the Membrane (M) Score. The relationship between CDH17 M Score and clinicopathological factors such as TNM staging, distant metastasis, and recurrence status was analyzed. The prognostic value of CDH17 M Score was determined by Kaplan–Meier curves and Cox regression analysis for overall survival (OS) and recurrence‐free survival (RFS). Comparison of M Score and pathologist visual scoring was made to assess the validity of software‐derived IHC scoring. CDH17 expression, as measured by M Score, was found to be significantly increased in tumor tissues compared to adjacent normal tissues, and its expression is associated with advanced staging and distant metastasis (normal vs. tumor, *p* = 0.0011; Stages IV vs. I–III, *p* < 0.0162; with vs. without metastasis, *p* = 0.0026; Mann–Whitney U test). Prognostic analysis revealed that high CDH17 M Score was associated with poor OS and RFS (OS, *p* = 0.0118; RFS, *p* = 0.0021; log‐rank test). Furthermore, multivariate Cox regression analysis identified that CDH17 M Score was an independent prognostic predictor for OS (HR = 2.296, 95% CI = 1.154–4.968, *p* = 0.0240) and RFS (HR = 2.489, 95% CI = 1.062–6.494, *p* = 0.0447).

**Conclusion:**

Increased CDH17 M Score was associated with advanced tumor staging and poor survival outcomes using an automated IHC system integrated with a digital image analysis software, highlighting CDH17 could serve as an independent prognostic marker for CRC patients.

## 1 Introduction

Colorectal cancer (CRC) is one of the most prevalent cancers worldwide, with up to 1 million deaths annually [1]. Despite the advances in understanding the molecular pathogenesis and mechanisms of CRC, there are currently no reliable predictors for recurrence risk or survival outcomes. This poses a major challenge in patient management, as approximately 30% of Stage II and over half of Stage III CRC patients experience a recurrence or a cancer within 5 years of initial treatment, with up to 18% and 40% mortality following the recurrence, respectively [2]. The high prevalence of disease recurrence highlights the early predictive monitoring as one of the major goals in patient management of CRC.

Cadherin 17 (CDH17) is a calcium‐dependent cell adhesion transmembrane glycoprotein that is responsible for maintaining tissue integrity and morphogenesis [3, 4]. It is a membrane protein expressed along the epithelia tissues of gastrointestinal (GI) tract, limited to the colon, small intestine, and pancreas at a regulated level in healthy individuals [5, 6]. Overexpression of CDH17 is widely reported in various GI cancers, including CRC [7, 8], gastric cancer [9, 10, 11], cholangiocarcinoma [12], hepatocellular carcinoma [13, 14, 15, 16], pancreatic cancer [17], neuroendocrine cancer [18], and esophageal cancer [19]. Inhibiting CDH17 has been shown to suppress tumor growth and metastasis [20], suggesting that CDH17 can be used as a biomarker to predict cancer prognosis and as a target for cancer intervention. The prognostic value of CDH17 has been studied in several GI malignancies. Notably, increased expression of CDH17 is associated with more aggressive tumor phenotypes and poor survival outcomes in cholangiocarcinoma and gastric cancers [12, 21]. In CRC, a significant association between high CDH17 level with liver metastasis and poor survival of the patients has been reported [7]. Nevertheless, a detailed analysis of CDH17 expression pattern and its predictive value in CRC recurrence risk remains limited.

The present study is aimed to investigate the expression pattern and prognostic impacts of CDH17 in CRC using an automated immunohistochemistry (IHC) platform in combination with a computer‐assisted scoring software. IHC is a widely used technique for confirming cancer diagnoses and can be used to identify characteristics of transformed tissues [22]. In this study, automation was incorporated into both the IHC procedures and the scoring analysis to reduce human error and minimize bias from visual perception, which are often encountered in manual methods [23]. The automated IHC machine ensures consistency in critical steps, such as antigen retrieval, reagent loading, incubation, and washing. As a result, it enhances the reliability and reproducibility of the staining process compared to manual methods. Additionally, the use of a computer‐assisted software for IHC analysis provides an objective and standardized approach for scoring the stained tissues. In this study, the software‐generated M Score, which integrates both the intensity and frequency of CDH17 membrane staining, was used to analyze a retrospective cohort of 150 CRC cases. Our findings revealed that a higher CDH17 M Score was associated with advanced tumor burden and poorer survival outcomes. These findings suggest that CDH17 is a potential biomarker for predicting CRC patient's prognosis and identifying patients who are at high risk of recurrence.

## 2 Materials and Methods

### 2.1 Clinical Samples and Data

This study is a retrospective study conducted on a cohort of 150 CRC patients, both male and female, who received pathological confirmation of their diagnosis at Queen Mary Hospital during the period from September 2014 to July 2022. Tumor and adjacent nontumor tissue specimens were collected after surgery and processed into formalin‐fixed, paraffin‐embedded (FFPE) tissue to perform subsequent analysis. Clinicopathological data for individual patients were retrieved for statistical analysis including their demographic data, tumor characterization (i.e., TNM staging, status of distant metastasis), and survival data (overall survival [OS] and recurrence‐free survival [RFS]). All patients had their diagnosis confirmed by a pathologist. The study was approved by the Institutional Review Board of our institution, and all tissues were collected with signed informed consent from patients.

### 2.2 CDH17 Automated Immunohistochemistry

#### 2.2.1 CDH17 Primary Antibody Selection and Optimization

The selection of CDH17 primary antibody and determination of optimal antibody dilution were performed on FFPE colorectal tissue sections. Five epitope specific anti‐CDH17 antibodies (Lic3, 10C12, 7C5, 9A6, and 8G5) targeted different CDH17 domains were evaluated for their binding activities in terms of CDH17 IHC staining pattern and intensity in paired adjacent nontumor and tumor colorectal specimens at a concentration of 1 ug/mL (Arbele Ltd., Hong Kong, China; Table S1). Cancer cell lines such as DLD1 (CDH17‐positive CRC) and SW480 (CDH17‐negative CRC) embedded in agar block were used as positive and negative controls, respectively (Figure S1). The selected antibody was titrated to optimize the contrast between positively stained tissue and nonspecific background staining with the highest primary antibody dilution. For initial titration, an antibody concentration from 0 to 2 ug/mL was tested and the best concentration with the optimal signal‐to‐background was selected for the subsequent automated CDH17 IHC assay.

### 2.3 Automated CDH17 IHC Assay

Automated IHC staining was performed according to the manufacturer protocol. In brief, sections for antigen retrieval were performed in an automated antigen retriever (GT‐R100, GeneTech, Shanghai, China) that followed the below steps and conditions: (1) prewarm the antigen retrieval buffer (GK800711‐D, GeneTech) to 60°C, (2) perform antigen retrieval at 98°C for 20 min, and (3) cool down the buffer to 80°C. The tissue slides were then transferred under running tap water for 5 min before being loaded onto the autostainer (GS2000, GeneTech).

The detection was performed using a ready‐to‐use secondary anti‐mouse HRP‐labelled antibodies (GK8007‐B, GeneTech), followed by signal development with DAB reagent (GK800711‐C1&2, GeneTech). All the reagents were diluted in PBS. Tris buffer supplemented with 0.05% Tween‐20 (TBS‐T) was used as washing buffer in between the staining steps. The staining protocol for CDH17 is detailed in Table 1. Upon completion of the CDH17 staining on the autostainer, the tissue slides were unloaded from the device and rinsed with running water for 5 min before counterstained with hematoxylin solution.

**TABLE 1:** Automated CDH17 IHC protocol.

Series	Steps	Reagents	Incubation (min)
1	Washing	TBS‐T	—
2	Blocking	Blocker (GK800711‐A, GeneTech)	10
3	Washing	TBS‐T	—
4	Primary antibody incubation	Lic3	50
5	Washing	TBS‐T	—
6	Secondary antibody incubation	Anti‐mouse HRP antibody (GK8007‐B, GeneTech)	20
7	Washing	TBS‐T	—
8	Coloration	DAB (GK800711‐C1&2, GeneTech)	5
9	Washing	TBS‐T	—

#### 2.3.1 Computer‐Assisted Image Analysis

Images of stained tissues were captured at 20× magnification using an Olympus microscope (BX63) coupled with a computer‐assisted IHC scoring system (GenAIs HiPath, Applied Spectral Imaging Inc). Five representative fields were selected for imaging in 24‐bit format and subsequent analysis, focusing on areas with well‐preserved tissue morphology while avoiding regions with necrosis or artifacts. On average, more than 10 000 cells were counted per sample, ensuring a robust and representative quantification of CDH17 expression. The CDH17 immunostaining was quantified by the M Score digital scoring method. The M Score is a semi‐quantitative measure of membrane staining incorporating both the staining intensity (*i*) and percentage of stained cells at each intensity level (Pi) with a calculated range from 0 to 50. The *i* values are graded as 0 (*lack of staining*), 1 (*weak staining*), 2 (*moderate staining*), and 3 (*strong staining*). The Pi values range from 0% to 100%. For each tissue section, the M Score was derived from the sum of *i* multiplied by the corresponding Pi and divided by six as the equation shown: M Score = [(0 × P0) + (1 × P1) + (2 × P2) + (3 × P3)]/(0 + 1 + 2 + 3). The default intensity threshold in the scoring system was used and guided by control slides when performing scoring analysis. The immunohistochemical quantification and analysis were made blinded to clinical data.

To evaluate the performance of computer‐assisted image analysis, whole slide images of 36 stained CRC cases were subjected to independent review by a pathologist. The pathologist assessed the percentage of positive cells for each *i* value, which was then used to calculate the M Score.

### 2.4 Statistical Analysis

Statistical analyses were performed using GraphPad Prism 9 software (GraphPad, CA, USA). Comparison of data between groups was performed using the Mann–Whitney U test. Survival analyses were conducted using the Kaplan–Meier method, and the log‐rank test was performed for survival comparisons between groups. Univariate and multivariate analysis was performed using a Cox proportional hazards model. Hazard ratios were reported with 95% confidence intervals. All *p* values < 0.05 were considered significant.

## 3 Results

### 3.1 Clinical Characteristics of Patients

In this retrospective study, a total of 150 patients with CRC were enrolled. These patients had a median age of 72 years (range 21–94 years) and a slight male predominance (60%). Tumor staging was based on the American Joint Committee on Cancer TNM system [24]. The majority of patients were presented with Stage II or III CRC (40.7% and 33.3%, respectively), with a low incidence of distant metastasis (8%). Details of clinicopathological characteristics of the included patients are listed in Table 2.

**TABLE 2:** Clinicopathological characteristics of CRC patients.

Variable	Number of patients *n* = 150
Age	
Median/range, years	72/21–94
Gender, *n* (%)	
Male	90 (60.0)
Female	60 (40.0)
Tumor differentiation, *n* (%)	
Well	7 (4.7)
Moderate	128 (85.3)
Poor	11 (7.3)
TNM stage, *n* (%)	
Stage 0	2 (1.3)
Stage I	25 (16.7)
Stage II	61 (40.7)
Stage III	50 (33.3)
Stage IV	12 (8.0)
Metastasis, *n* (%)	
Yes	12 (8.0)
No	138 (92.0)
Lymph node involvement, *n* (%)	
Yes	60 (40.0)
No	90 (60.0)

### 3.2 Expression Pattern of CDH17 in CRC

The expression levels of membrane CDH17 in CRC tissues were evaluated by IHC, and the quantification was performed using a digitally generated M Score. To optimize staining results, five different clones of in‐house monoclonal antibodies against different domains of CDH17 were tested. The results indicated that the Lic3 antibody at a concentration of 1 ug/mL produced the best staining outcomes, with a high signal‐to‐background ratio (Figure S1). Consequently, the Lic3 antibody at a concentration of 1 ug/mL was employed for the IHC staining. To assess the accuracy of the computer‐assisted image analysis of CDH17 expression, 36 stained CRC cases were reviewed by a pathologist to generate manually calculated M Scores. Correlation analysis showed a strong positive correlation between the digital M Score and pathologist‐calculated M Score (*R* = 0.7257, *p* < 0.001; Figure S2 and Table S2), suggesting that the computer‐assisted analysis is a reliable and accurate measure of tissue CDH17 expression.

**FIGURE 1:**
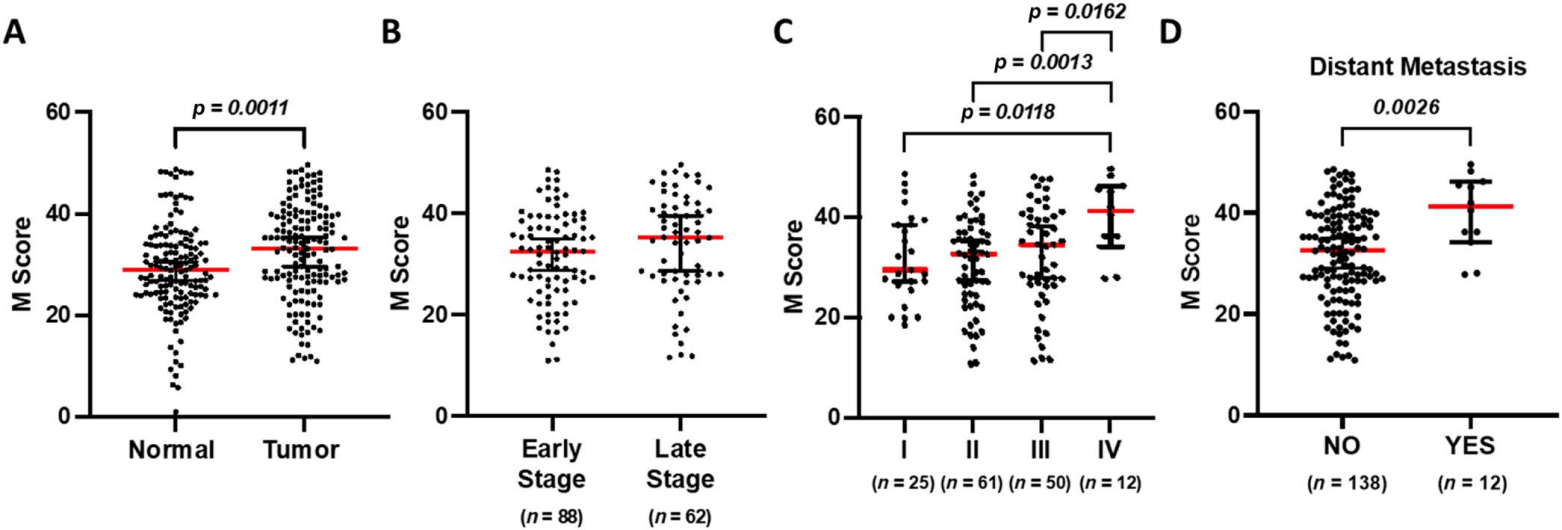
Retrospective analysis of 150 colorectal cancer (CRC) cases. (A) Comparison of M Scores between paired adjacent normal tissue and CRC tumor tissue. (B) Comparison of M Scores between tumor tissue from early‐stage (I and II) and late‐stage (III and IV) CRC. (C) Comparison of M Scores among tumor tissue from Stages from I to IV of CRC. (D) Comparison of M Scores between tumor tissue from metastatic and nonmetastatic CRC.

Analysis of M Score demonstrated a significantly higher expression level of CDH17 in tumor tissues compared to adjacent normal tissues (*p* = 0.0011. Figure 1A), with median CDH17 M Scores of 33.15 and 28.8, respectively. The lowest M Score found in tumor tissue was 10.9. A trend of higher CDH17 M Score was found in late‐stage CRC (III and IV) compared to early‐stage CRC (I and II) (*p* = 0.0806; Figure 1B). Furthermore, it was observed that Stage IV CRC exhibited the highest CDH17 expression level among all stages of CRC, as evidenced by its significantly higher M Score value (IV vs. I, *p* = 0.0118; IV vs. II, *p* = 0.0013; IV vs. III, *p* = 0.0162; Figure 1C). In addition, CRC cases with metastasis displayed an increased CDH17 M Score compared to nonmetastatic CRC (*p* = 0.0026; Figure 1D), suggesting a more pronounced CDH17 expression in cases at a more advanced stage.

### 3.3 Prognostic Analyses of CDH17 Expression in CRC Patients

**FIGURE 2:**
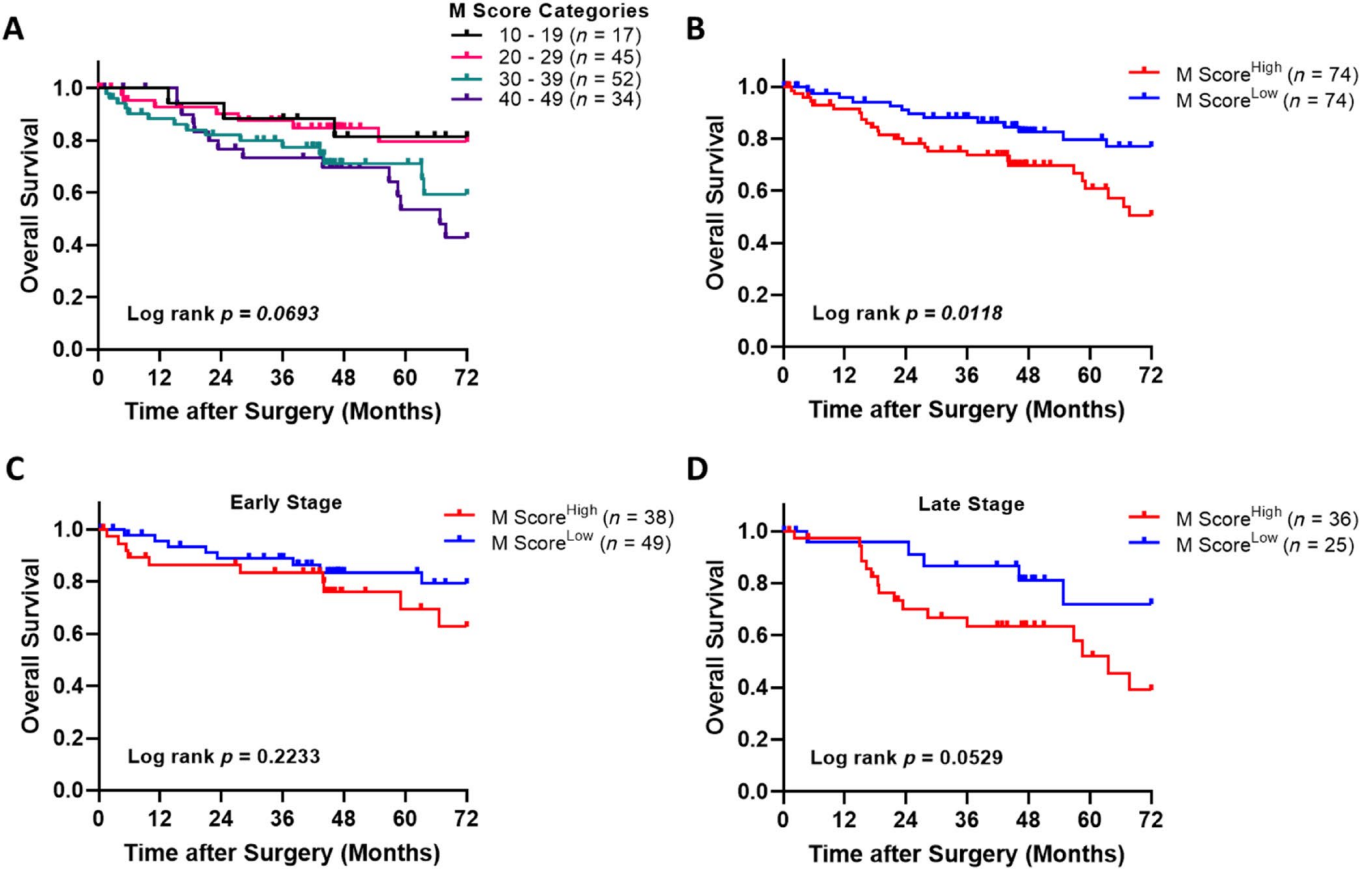
Kaplan–Meier survival analysis of the prognostic impact of M Score on overall survival. (A) Overall survival curve of patients by M Score categories. (B) Overall survival curve of patients categorized as high M Score and low M Score. (C) Overall survival curve of patients with early‐stage CRC categorized by high and low M Score. (D) Overall survival curve of patients with late‐stage CRC categorized by high and low M Score. The cut‐off value for high M Score = 33.15.

The prognostic significance of CDH17 M Score on OS and RFS was investigated in CRC patients. Patients were categorized into four groups based on their tumor tissue CDH17 M Score values. A noticeable trend of decreasing OS was observed with increasing M Score intervals (*p* = 0.0693, Figure 2A). Although this trend was near‐significant, it suggests that higher CDH17 expression may be associated with poorer outcomes. Notably, patients in the M Score 30–39 and 40–49 groups showed a distinctively shorter OS. When the patients were dissected into M Score^High^ and M Score^Low^ groups using the median M Score value of 33.15 as the cut‐off, patients with a high CDH17 M Score showed significantly poorer OS (*p* = 0.0118, Figure 2B), further supporting the prognostic impact of CDH17 expression in CRC. This trend was consistent in late‐stage CRC (*p* = 0.0529, Figure 2D) indicating that high CDH17 expression might serve as a stronger predictor of poorer prognosis in advanced disease stages. In contrast, the prognostic value of CDH17 in early‐stage CRC was less apparent (*p* = 0.2233, Figure 2C), suggesting that CDH17 may have a more limited role in predicting outcomes in early stages of the disease.

**FIGURE 3:**
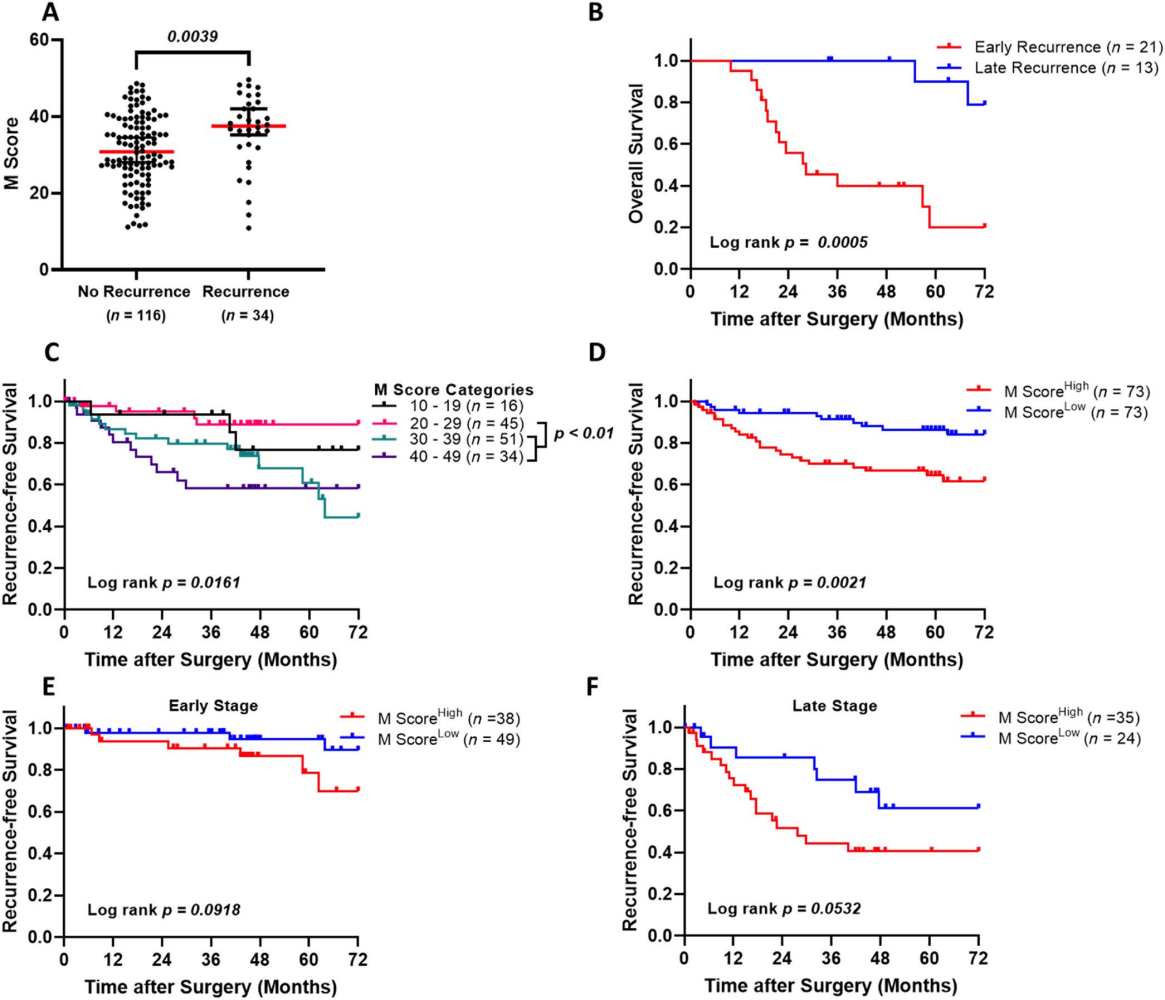
Kaplan–Meier survival analysis of the prognostic impact of M Score on recurrence‐free survival (RFS). (A) Comparison of M Scores between tumor tissue from CRC patients with and without recurrence. (B) Overall survival curve of patients categorized by early (< 2 years) and late recurrence (≥ 2 years). (C) RFS curve of patients by M Score categories. (D) RFS curve of patients categorized as high M Score and low M Score. (E) RFS curve of patients with early‐stage CRC categorized by high and low M Score. (F) RFS curve of patients with late‐stage CRC categorized by high and low M Score. The cut‐off value for high M Score = 33.15.

Patients with recurrence had significantly higher CDH17 M Scores compared with patients without recurrence (*p* = 0.0039; Figure 3A), indicating that elevated CDH17 expression is associated with a higher risk of disease relapse. Furthermore, patients with early recurrence, which is defined as the relapse time within 24 months after the surgery [25, 26], had significantly shorter OS compared to those with late recurrence (*p* = 0.0005; Figure 3B). Increasing M Score intervals were associated with shorter RFS (*p* = 0.0161, Figure 3C), with significantly lower RFS observed in the M Score 30–39 and 40–49 groups compared to the 20–29 group (*p* < 0.01). Similarly, patients in the M Score^High^ group exhibited poorer RFS (*p* = 0.0021; Figure 3D). While clear separation of RFS curves between the high and low M Score groups was evident in both early‐ and late‐stage CRC (Figure 3E,F), with more pronounced separation in late‐stage disease (*p* = 0.0532), the difference in RFS between M Score groups did not reach statistical significance. The overall findings suggest that patients with higher CDH17 M Scores not only had a higher likelihood of recurrence but also experienced worse RFS.

Univariate and multivariate analysis using a Cox proportional hazard model further identified CDH17 expression as an independent prognostic predictor for both the postoperative OS (HR [95% CI]: 2.296 [1.154–4.968], *p* = 0.0240; Table 3) and RFS (HR [95% CI]: 2.489 [1.062–6.494], *p* = 0.0447; Table 3) in CRC patients.

**TABLE 3:** Univariate and multivariate Cox regression analyses of OS and RFS in colorectal cancer patients.

Variables		Overall survival					Recurrence‐free survival			
		Univariate analysis	Multivariate analysis				Univariate analysis	Multivariate analysis		
		*p*	HR	95% CI	*p*		*p*	HR	95% CI	*p*
Gender	Male versus female	0.4154					0.8272			
Age at diagnosis (years)	< 72 versus ≥ 72	***0.0051***	2.558	1.324–5.246	***0.0069***		0.6390			
Location	Colon versus rectum	0.7016					***0.0087***	2.118	0.920–5.279	0.0875
TNM Stage	I + II versus III + IV	0.0665					***< 0.0001***	28.20	2.758–301.8	***0.0035***
Lymph node status	N0 versus N1 + 2	0.1734					***0.0003***	0.1923	0.022–1.628	0.1068
Distant metastasis	M0 versus M1	0.1297					***0.0029***	0.5254	0.083–1.869	0.3936
Differentiation	Well + moderately versus poorly + undifferentiated	0.7508					0.9346			
CDH17 expression^a^	Low versus high	***0.0144***	2.210	1.155–4.445	***0.0199***		***0.0037***	2.489	1.062–6.494	***0.0447***

^a^ Cut‐off M Score = 33.15.

## 4 Discussion

A reliable prognostic biomarker for CRC is currently lacking. CDH17 has been shown to have a prognostic role in GI cancers [7, 12, 21]. In this study, the expression pattern and prognostic significance of CDH17 in CRC tissue was investigated. The results demonstrated that overexpression of CDH17, as measured by the M Score, was associated with advanced cancer staging and was identified as an independent prognostic factor for OS and RFS. Overall findings suggest that CDH17 tissue expression may be a useful prognostic biomarker for CRC patients.

Previous studies have shown that CDH17 is associated with tumor progression and aggressiveness in various types of GI cancers, including CRC [7], hepatocellular carcinoma [13, 14, 16], gastric cancer [27, 28], and cholangiocarcinoma [12]. CDH17 immunopositivity has been commonly reported in CRC, and the positive rates were as high as > 95% in colorectal neoplasm [29, 30]. In line with these findings, the present study found that CDH17 was aberrantly overexpressed in CRC tissue with a 100% positivity rate. The increased CDH17 expression was more prominent in advanced stage CRC and in metastasis, indicating a potential role of CDH17 in CRC progression and aggressiveness. Survival curve analyses further revealed the prognostic implications of CDH17 for both RFS and OS in CRC patients, with high M Score values associated with poorer survival outcomes and predictive for disease recurrence. Multivariate analysis also showed that high CDH17 expression was an independent predictor of OS and RFS, with more than a 2‐fold hazard ratio of predicting patients with worse survival outcomes. These results are concordant with a retrospective study that examined the prognostic significance of CDH17 in cholangiocarcinoma [12]. The study also identified CDH17 expression as an independent prognostic factor for OS and demonstrated a superior prognostic performance of CDH17 compared to the routinely used biomarker CA19‐9 and TNM staging system in predicting patients' survival outcomes.

The mechanisms by which CDH17 overexpression promotes CRC progression are not fully understood. However, several studies have suggested that CDH17 may act through the integrin, NFκB, and Wnt signaling pathways. CDH17 has been shown to regulate integrin signaling and induce downstream Ras activation, which promoted cell proliferation in gastric cancer and CRC [31]. In an in vivo study, knockdown of CDH17 in gastric cancer cells suppressed tumor growth and lymphatic metastasis [32]. This was associated with the downregulation of NFκB target genes, including VEGF‐C and MMP‐9, probing the involvement of the NFκB in driving tumor progression. Another study showed that targeting CDH17 inhibited tumor growth through the inactivation of the Wnt signaling pathway in hepatocellular carcinoma [14]. These findings support the hypothesis that CDH17 plays a pivotal role in cancer development and hence is a promising prognostic biomarker. Further mechanistic studies are needed to elucidate the specific role of CDH17 in CRC tumorigenesis.

The present study employed a computer‐assisted image analysis to quantitate CDH17 expression in CRC tissues, offering a more standardized and objective approach compared to the traditional manual scoring methods [33, 34]. The M Score digital scoring method combines staining intensity, categorized on a 4‐point scale (from 0 to 3+), with the percentage of positive cells, offering a comprehensive evaluation of CDH17 protein expression. The performance of this computer‐assisted image analysis was validated by comparing the results with independent pathologist reviews, ensuring the reliability and reproducibility of the current findings. The use of the 4‐point intensity scale in the M Score helps maintain consistency by reducing potential noise arising from variations in image quality or sample preparation. However, continuous intensity measurements could provide a finer resolution, potentially enhancing the precision of scoring. Future studies may explore the possibility of incorporating continuous intensity measurements to further refine and improve the scoring system. In addition, considering the heterogeneity of CRC, one of the main limitations of our study is the localized patient demographics and relatively small population size. Notably, the number of patients within the M‐score 10–19 group is limited, which may affect the statistical power and generalizability of our findings. Further investigations in larger and diverse prospective cohorts are warranted to confirm the prognostic significance of CDH17 expression in CRC. The inclusion of multicentered recruitment of patients in future studies would strengthen the generalizability and validity of the findings.

Overall, our study contributes to the understanding of CDH17 expression in CRC and its implications for patient prognosis. The findings suggest that CDH17 may serve as a potential prognostic biomarker in CRC, which helps in patient stratification and treatment decision‐making.

## Author Contributions

L.N., J.M.L., D.A.W., and D.C.‐C.F. conceived and designed the experiments. N.M.A. and P.L. performed the experiments. S.S. and W.S.Y. analyzed the data. L.N., J.M.L., and D.C.‐C.F. contributed reagents/materials/analysis tools. S.S. and W.S.Y. wrote the paper.

## Ethics Statement

The study was approved by the Institutional Review Board of the University of Hong Kong/Hospital Authority Hong Kong West Cluster (reference number: UW 21–114), and all tissues were collected with signed informed consent from patients.

## Consent

The authors have nothing to report.

## Conflicts of Interest

All authors declare no conflicts of interest.

## Supporting information


**Table S1.** Different clones and isotypes of primary antibody against CDH17.
**Figure S1.** (A) Representative IHC images and corresponding M Score of CRC and adjacent normal tissues stained with five different clones of primary antibody against CDH17. (B) Representative IHC images and corresponding M score of CRC and adjacent normal tissues stained with different working concentrations of Lic3 antibody. (C) Representative IHC images of the CDH17 negative (SW480) and positive (DLD1) cell line controls. Magnification: ×20.
**Figure S2.** Linear regression plot of Digital Image Analysis derived M Score versus pathologist calculated M Score. The M score data are listed in Table S1.

## Data Availability

The data that support the findings of this study are not openly available due to reasons of sensitivity and are available from the corresponding author upon reasonable request.
